# A Scanning Electron Microscope Study on the Effect of an Experimental Irrigation Solution on Smear Layer Removal 

**Published:** 2014-03-08

**Authors:** Vahid Zand, Hadi Mokhtari, Mehrdad Lotfi, Saeed Rahimi, Aydin Sohrabi, Sina Badamchi Zadeh, Hanieh Mojaver Kahnamooie, Pardis Tehranchi

**Affiliations:** a * Dental and Periodontal Research Center, Department of Endodontics, Dental School, Tabriz University of Medical Sciences, Tabriz, Iran; *; b * Department of Endodontics, Dental School, Tabriz University of Medical Sciences, Tabriz, Iran; *; c * Dental and Periodontal Research Center, Department of Orthodontics, Dental School, Tabriz University of Medical Sciences, Tabriz, Iran;*; d * Department of Prosthodontics, Dental School, Hamedan University of Medical Sciences, Hamedan, Iran; *; e * Department of Endodontics, Faculty of Dentistry, Hamedan University of Medical Sciences, Hamedan, Iran; *; f * Dental and Periodontal Research Center, Department of Operative Dentistry, Faculty of Dentistry, Tabriz University of Medical Sciences, Tabriz, Iran*

**Keywords:** EDTA, NaOCl, Papain, Scanning Electron Microscopy, SEM, Smear Layer

## Abstract

**Introduction:** The aim of this *in vitro* study was to evaluate the effect of an experimental irrigation solution, containing two different concentrations of papain, Tween 80, 2% chlorhexidine and EDTA, on removal of the smear layer. **Methods and Materials:** Thirty-six single-rooted teeth were divided into two experimental groups (*n*=12) and two positive and negative control groups of six. The canals were prepared with BioRaCe instruments up to BR7 (60/0.02). In group 1, canals were irrigated with a combination of 1% papain, 17% EDTA, Tween 80 and 2% CHX; in group 2, canals were irrigated with a combination of 0.1% papain, 17% EDTA, Tween 80 and 2% CHX. In group 3 (the negative control), the canal was irrigated with 2.5% NaOCl during instrumentation and at the end of preparation with 1 mL of 17% EDTA was used; in group 4 (positive control), normal saline was used for irrigation. The amount of the remaining smear layer was quantified according to Hulsmann method using scanning electron microscopy (SEM). Data was analyzed by the Kruskal-Wallis and Mann-Whitney tests. **Results:** Two-by-two comparisons of the groups revealed no significant differences in terms of smear layer removal at different canal sections between the negative control group (standard regiment for smear layer removal) and 1% papain groups (*P*<0.05). **Conclusion:** Under the limitations of the present study, combination of 1% papain, EDTA, 2% chlorhexidine and Tween 80 can effectively remove smear layer from canal walls.

## Introduction

During canal debridement and shaping, organic and inorganic remnants originating from the pulp and dentine, adhere to the canal walls, forming an amorphous or irregular layer referred to as the smear layer [1, 2]. There is controversy over preservation or removal of the smear layer. Despite the paucity of clinical data regarding treatment success, a review article emphasized that the smear layer may contain bacteria and necrotic tissue and thus should be removed [2]. It is stated that this layer forms a barrier between the filling material and sound dentin that inhibits the penetration of resin tags into dentinal tubules and thus increases the microleakage with commonly used sealers, and decreases the bond strength of resin-based materials. It also prohibits the canal irrigants from reaching the microorganisms hiding within the tubular spaces [2-7].

The most common irrigation regiments for the removal of the smear layer includes sodium hypochlorite (NaOCl) at various concentrations to remove the organic component of the smear layer and complementing its action by means of chelating agents such as 17% ethylenediaminetetraacetic acid (EDTA) to remove the inorganic components [6]. NaOCl has superb tissue dissolving properties and antibacterial activity, which make it the irrigant of choice for the root canal treatment of necrotic teeth; however, it will have widespread cytotoxicity if it enters the periapical tissues through the apical foramen, resulting in the necrosis and inflammation of periapical tissues [8, 9].

In the present study, to overcome the toxicity of NaOCl, an attempt was made to use an enzyme-based agent to remove the smear layer. The used irrigant was a combination of a protease called papain and a lipase known as Tween 80, along with 2% chlorhexidine gluconate as an antibacterial agent [10] and EDTA for the removal of the inorganic component [11]. Papain is an enzyme from the cysteine protease family, which belongs to a family of related proteins with a wide variety of activities, including endopeptidases, aminopeptidases, dipeptidyl peptidases and enzymes with both exo/endo-peptidase activity [12, 13]. Papain has been used in pediatric dentistry in a gel called Papacarie (Formula e Ação, São Paulo, Brazil) for chemomechanical removal of the carious lesions without the use of burs. The material is a gel product consisting of papain, toluidene blue, and chloramine which is able to remove carious lesions in 8 min [14, 15]. *In vitro* studies have demonstrated acceptable biocompatibility and genotoxic and cytotoxic safety [16, 17].

The aim of the present *in vitro* study was to evaluate the effect of an experimental irrigation solution containing papain at two different concentrations, Tween 80, 2% chlorhexidine and 17% EDTA, on the removal of the smear layer in coronal, middle and apical sections.

## Methods and Materials

A total of 36 freshly extracted human anterior single-rooted teeth were used for this study. Radiography was taken from all the teeth to ensure the presence of a single canal, a mature apex, and absence of calcification, resorption or endodontic obturation. The degree of canal curvature was determined using Schneider’s method [18] and teeth with the radii of curvature less than 5° were selected. The samples were kept in 0.1% thymol solution and stored at 37° C in an incubator for 72 h. Each tooth was decoronated at 15 mm from the anatomic apex and the pulp tissue if existed, was removed with a barbed broach (Dentsply, Maillefer, Ballaigues, Switzerland). Working length (WL) was established at 1 mm short of the apical foramen with a #10 K-file (Dentsply Maillefer, Ballaigues, Switzerland) and the anatomic diameter of the canal at WL was also determined by the first binding instrument among the successively larger K-type files introduced to the canal. The anatomic diameter of the root canal was recorded when the instrument showed resistance against removal from the working length. All the teeth with a canal diameter greater than a #40 file at WL were discarded and replaced with others. The apical parts of the roots were put inside polyvinylsiloxane impression material during instrumentation.

The teeth were then randomly divided into two experimental groups (*n*=12) and two positive and negative control groups, each one containing 6 teeth. Instrumentation was performed by means of BioRaCe rotary instruments (FKG; Dentaire, La-Chaux-de-Fonds, Switzerland) according to manufacturer’s instructions with a crown-down technique and the following sequence: BR0 (25/0.08) for coronal pre-flaring and then BR1 (15/0.05), BR2 (25/0.04), BR3 (25/0.06), BR4 (35/0.04), BR5 (40/0.04), BR6 (50/0.04), and finally BR7 (60/0.02) for apical preparation. These instruments were installed on an 8:1 reduction handpiece powered by a torque-limited electric motor (TCM Motor 3000; Novage, Konstanz, Germany) with the rotational speed of 500 rpm.

During instrumentation of the samples and after each instrument, a 10 mL solution containing a combination of 1% papain, 17% EDTA (Merck, Darmeshtadt, Germany), Tween 80 and 2% chlorhexidine gluconate (Consepsis Ultradent Products, Inc., South Jordan, UT, USA) was used for group 1, and a 10 mL solution containing 0.1% papain, 17% EDTA, Tween 80 and 2% chlorhexidine gluconate was administered for group 2.

In the negative control group (group 3), 10 mL of 2.5% NaOCl was used after each instrument and at the end of preparation, 1 mL of 17% EDTA was used.

In the positive control group (group 4), 10 mL of normal saline was used for root canal irrigation during instrumentation. In all the groups, the irrigants were divided equally between each instrument during and after instrumentation and a #10 K-file was inserted 1 mm beyond the WL to maintain the apical patency between instruments. The irrigating solutions were delivered via a sterile 30-gauge ProRinse needle (Dentsply, Tulsa Dental, Johnson City, TN, USA), which penetrated within 1 to 2 mm of the WL. All the root canals were then irrigated with 5 mL of distilled water as a final rinse and dried with sterile paper points.

***Scanning electron microscopy (SEM)***

Two longitudinal grooves were prepared on the buccal and lingual surfaces of each root using a diamond disc without penetration into the canal. The roots were then split into two halves with a chisel. For each root, the half containing the most visible part of the apex was preserved and coded. The coded specimens were then mounted on metallic stubs, gold-sputtered, and examined under SEM (DSM 940 A; Vega Tescan, USA) at ×200, ×500 and ×1000 magnifications. The existence of smear layer in coronal, middle and apical sections were scored according to method reported by Hulsmann *et al.* at ×1000 magnification: (1) no smear layer, orifice of dentinal tubules patent; (2) small amount of smear layer, some open dentinal tubules; (3) homogenous smear layer along almost the entire canal wall, only very few open dentinal tubules; (4) the entire root canal wall covered with a homogenous smear layer, no open dentinal tubules; and (5) a thick homogenous smear layer covering the entire root canal wall [19].

**Table 1:** Mean (SD) of smear layer in different groups

	**Negative control**	**1% Papain**	**0.1% Papain**	**Positive control**
**Coronal third**	1.00 (0.00) ^a^	1.08 (0.28) ^a^	1.33 (0.64) ^a^	4.50 (0.80) ^b^
**Middle third**	1.33 (0.49) ^a^	1.67 (0.87) ^a^	2.50(1.64) ^a,b^	3.33 (1.78) ^b^
**Apical third**	1.83 (1.11) ^a^	2.42 (1.35) ^a^	4.00 (1.25) ^b^	4.00 (1.60) ^b^

*Similar letters indicate non-significant differences between groups in the same section.*

**Table 2:** Mean (SD) of smear layer in different sections

	**Negative control**	**1% Papain**	**0.1% Papain**	**Positive control**
**Coronal third**	1.00 (0.00)^2^	1.08 (0.28)^3^	1.33 (0.64)^3^	4.50 (0.80)^1^
**Middle third**	1.33 (0.49)^1,2^	1.67 (0.87)^2^	2.50 (1.64)^2^	3.33 (1.78)^1^
**Apical third**	1.83 (1.11)^1^	2.42 (1.35)^1^	4.00 (1.25)^1^	4.00 (1.60)^1^

*Similar digits indicate non-significant differences between different levels in the same group*

All root canal preparations were completed by one operator, whereas the SEM evaluations were performed by three other examiners who were blind to the experimental groups. Every examiner made scoring for several areas and then took the average. Scores 1 and 2 were considered suitable scores [19, 20]. Data were analyzed by the Kruskal-Wallis test and Mann-Whitney U ran sum test for pair-wise comparisons. The level of statistical significance was set at 0.05.

## Results

Root canal walls absolutely free of smear layer were not observed with any of the irrigation solutions (Table 1). The Kruskal-Wallis test revealed statistically significant differences in all the canal sections (coronal, middle and apical thirds) between all the irrigation solutions (*P*<0.001, *P*=0.013, *P*<0.001, respectively). Two-by-two comparisons of the groups under study did not reveal any significant differences in the three canal sections between the negative control group and the group in which a solution containing 1% papain was used (*P*=0.704, *P*=0.436 and *P*=0.295, respectively).

**Figure 1:**
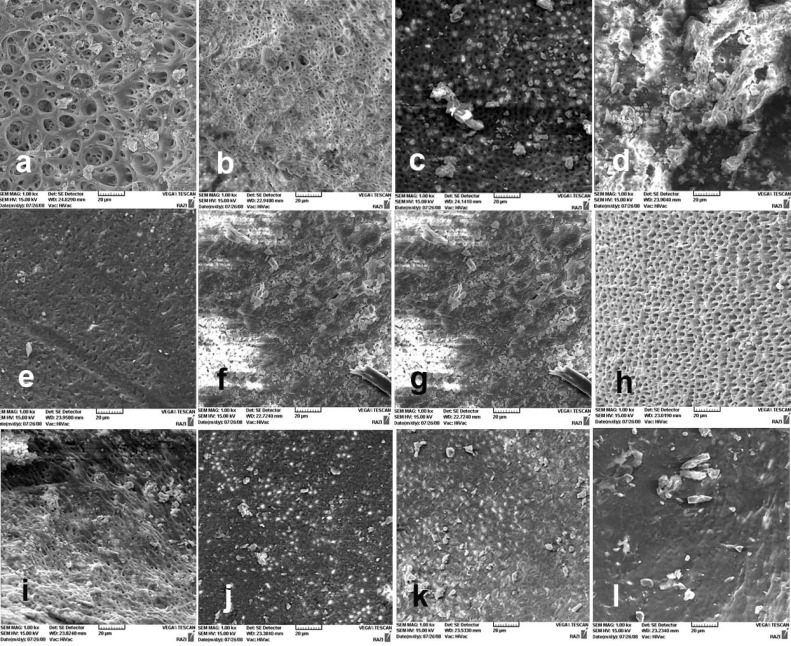
*A)* The coronal third of the canal wall after using 1% papain solution; almost no smear layer is remained. Orifice of dentinal tubules are patent (score 1); *B)* The middle third of the canal wall after using 1% papain solution (score 1); *C)* The apical third of the canal wall after using 1% papain solution, small amount of smear layer, some open dentinal tubules are visible (score 2); *D)* The coronal third of the prepared canal wall after using 0.1% papain solution; note the homogenous smear layer along almost the entire canal wall. Only very few open dentinal tubules are present (score 3); *E)* The middle third of the prepared canal wall after using 0.1% papain solution; the entire root canal wall is covered with a homogenous smear layer and open dentinal tubules are absent (score 4); *F)* The apical third of the prepared canal wall after using 0.1% papain solution; a thick, homogenous smear layer covering the entire root canal wall is evident (score 5). The canal wall in negative control sample; *G)* The clean canal wall in the coronal third of the prepared canal (score 1); *H)* The clean canal wall in the middle third of the prepared canal (score 1); and *I)* The canal wall in the apical third (score 2). The canal wall in positive control sample; *J)* The coronal third of the prepared canal (score 5); *K)* The middle third of the prepared canal (score 4); and *L)* The apical third of the prepared canal (score 5)

Statistically significant differences were observed in all the canal sections between solutions (Figure 1) containing 1% and 0.1% papain (*P*=0.111, *P*=0.103 and *P*<0.001, respectively). There were statistically significant differences between the positive control group and all the experimental groups in three canal sections (*P*<0.05).

## Discussion

Different chemical formulations of irrigants might have different reactions on the pulp and periapical tissues, leading to tissue necrosis [21, 22]. In order to eliminate the smear layer, the irrigation solution needs to be able to remove both its organic and inorganic components. In a protocol suggested by Torabinejad *et al.*, a mixture of tetracycline, acid and detergent (MTAD) is used during canal preparation [23]. Despite a decrease in NaOCl concentration in the above-mentioned protocol, its potential toxicity during canal preparation is still a clinical dilemma [8, 9]. In the present study, an attempt was made to use an enzyme-based compound to remove the organic component of the smear layer.

The compound used consisted of a protease called papain and a lipase called polyoxyethylene sorbitan mono-oleate (Tween 80), which have already been included as a surfactant in MTAD [24, 25]. Two different concentrations of papain were used in the experimental groups, *i.e.* 1% (in group 1) and 0.1% (in group 2). Since the highest solubility of papain in water is 0.1%, this concentration of papain was used to evaluate its low concentration.

Although FDA has voiced doubts regarding the safety of papain, the majority of reports about the risks of local application of papain are related to the eyes and skin, resulting from long-term exposure to papain [26]. However, there is still controversy over this issue [27]. In addition, there are reports of cytotoxicity, genotoxicity and risks associated with the direct use of NaOCl on live tissues. However, it has been reported that papain lacks cytotoxic and genotoxic effects [28]. In the present study, papain was used at low concentrations as an irrigation solution for a few minutes. 

However, it appears that further studies are required regarding the safety of papain at low concentrations. Because of the root encasement in the bone socket during clinical cleaning and shaping [29, 30], the canal behaves as a closed-end channel, which results in gas entrapment at its closed end [30], producing a vapor lock effect during irrigant delivery [31, 32]. In the present study, all the roots were embedded in polyvinylsiloxane to simulate a closed-end canal system, and the apical patency was maintained to minimize the presence of gas bubbles in big canals [32, 33].

In the present study, the teeth in all the experimental groups were balanced with respect to the apical diameter of the root canal [34], and an identical sequence of rotary files were used in all the specimens for canal preparation. Rotary files produce a significant amount of smear layer [35-38].

The results of the present study demonstrated that papain had proteolytic effects in all of the three canal sections, which is in line with the results of other studies [33, 34]. Studies have shown the proteolytic action of papain in cartilage of the lung parenchyma which is composed of collagen fibrils and proteoglycans, like the dentin matrix [33]. Research has shown that papain promotes the digestion of proteoglycans of the extracellular matrix, such as decorin and biglycan [33]. Furthermore, another study has shown that intact nonmineralized type I collagen fibrils are partially degraded by a papain gel in dentin specimens [31]. In addition, intact mineralized dentin had reduced mechanical properties after treatment with the papain gel, presumably due to the effect of the protease on dentin proteoglycans and possibly on the mineralized collagen fibrils as well [34]. These issues can be the subject of future studies. Further investigations should be conducted to evaluate the probable adverse effects of these experimental irrigants on root dentine.

## Conclusion

Within the limitations of this *in* *vitro* study, a combination of 1% papain, 17% EDTA, 2% CHX and Tween 80 can be effective in removing the smear layer from the canal walls.
